# *Listeria monocytogenes* infection associated with alemtuzumab – - a case for better preventive strategies

**DOI:** 10.1186/s12883-017-0848-8

**Published:** 2017-04-04

**Authors:** Trygve Holmøy, Hedda von der Lippe, Truls Michael Leegaard

**Affiliations:** 1 Department of Neurology, Akershus University Hospital, Lørenskog, Norway; 2 Institute of Clinical Medicine, University of Oslo, Oslo, Norway; 3 Department of Infectious Diseases, Akershus University Hospital, Lørenskog, Norway; 4 Department of Microbiology, Akershus University Hospital, Lørenskog, Norway

**Keywords:** Multiple sclerosis, Treatment, Alemtuzumab, Adverse events, *Listeria monocytogenes*

## Abstract

**Background:**

The mortality of septicaemia, meningitis and encephalitis caused by *Listeria monocytogenes* is 20–40%. Twenty-one cases of invasive listeriosis associated with alemtuzumab, including at least 16 in patients with multiple sclerosis, have been published or reported to the World Health Organization Case Safety Reports Database. Three cases were fatal, including at least one patient treated for multiple sclerosis in 2016.

**Case presentation:**

We report a patient with multiple sclerosis who developed pyrexia, nausea and abdominal discomfort few hours after the third and last infusion of her second alemtuzumab cycle. An infusion related reaction was suspected. The patient had however eaten soft cheese and raw sausage 3 days prior to treatment, and *L. monocytogenes* septicaemia was diagnosed based on positive blood cultures.

**Conclusion:**

Listeriosis associated with alemtuzumab is a potentially fatal condition that can mimic an infusion related reaction. As in most other previously reported cases symptoms started rapidly after the last infusion, suggesting that the patient already carried the bacteria prior to the alemtuzumab infusions. The summary of product characteristics recommends patients to avoid foods associated with listeria at least 1 month after treatment. This recommendation should include also the last weeks prior to treatment.

## Background

Listeriosis is caused by the Gram positive bacteria *Listeria monocytogenes*, and is usually contracted from unpasteurized dairy products, raw fish and meat, or products made from pasteurized products contaminated with *L. monocytogenes* after production, like soft cheeses. Immunocompetent persons rarely develop severe symptoms, whereas people with defective cellular immunity may develop septicaemia, meningitis or encephalitis, with a mortality rate ranging from 20 to 40% [1, 2].

The importance of listeriosis associated with alemtuzumab in multiple sclerosis (MS) has recently been underscored by a fatal case not yet published, but that has been reported to VigiBase©, the World Health Organization international database of suspected adverse drug reactions [3] and to Sanofi Genzyme (Sanofi Genzyme, data on file). The current case history highlights that listeriosis must be considered in patients who develop pyrexia shortly after treatment with alemtuzumab, even in the absence of meningism. It also suggests that the Summary of Product Characteristics (SPC) should be revised to minimize the risk of this potentially fatal complication.

## Case presentation

The patient is a woman in her early fifties. She was diagnosed with MS after a sensory attack in the left shoulder in 2008 and a sensorimotor attack in the right leg in 2013. She was treated with interferon beta 1a from April 2013, and with fingolimod from September 2013 after a motor attack in the left leg from which she recovered partially. Treatment was changed again to natalizumab in January 2014 when macula edema was suspected. She remained clinically and radiologically stable until natalizumab was terminated in the beginning of June 2015, after she tested positive for John Cunningham virus. Alemtuzumab was started at the end of July 2015. During the first cycle (12 mg for 5 days) she had transient sinus bradycardia down to 30 beats per minute but no other adverse events.

The patient remained clinically stable with an expanded disability status scale (EDSS) score at 2.5 until the second cycle (12 mg alemtuzumab preceded by 1000 mg methylprednisolone, 12 mg cetrizine and 1000 mg paracetamol for three consecutive days) in July 2016. Except for transient bradycardia there were no immediate adverse reactions, but some hours after the last infusion of alemtuzumab she became sick with nausea and fever up to 40 °C. At admission to hospital she was awake and did not have neck stiffness or other focal signs except abdominal discomfort and mild headache. She was febrile (39.5 C) and clinically dehydrated but normotensive. C-reactive protein was 180, lymphocytes were below the detection limit but the number of granulocytes was normal. As she did not have new neurological symptoms, neither detailed neurological examination, brain imaging nor lumbar puncture were performed. Four out of four blood cultures were positive for *L. monocytogenes* (confirmed by 16S RNA sequencing) which was susceptible to trimethoprim-sulphamethoxazole, ampicillin, erythromycin, meropenem and penicillin. She recovered rapidly and completely upon treatment with ampicillin and trimethoprim-sulphamethoxazole.

## Discussion

To our knowledge, this is the 22nd case of listeriosis associated with alemtuzumab reported so far, either in the literature or to the WHO database VigiBase [3–6]. Including the present case, at least 16 of these have occurred in patients treated for MS (Table 1). Until January 2017 approximately 11,500 MS patients have been treated with alemtuzumab (Sanofi Genzyme, data on file), indicating that the risk of listeriosis is in the range of 0.1%. It should be noted that only one case is reported outside Europe (Australia). This could indicate that this complication of alemtuzumab might be under-reported in some areas, as the general prevalence of listeriosis in North America is comparable to that in Europe [2].

**Table 1:** Characteristics of previously reported cases of listeriosis associated with alemtuzumab reported until February March 3, 2017

Source (reference)	Type of listeriosis	Gender	Indication	Number of infusions	Days from first infusion to onset	Outcome
VigiBase 2017 (3)	Meningitis	Female	Multiple sclerosis	5	Unknown	Unknown
VigiBase 2016 (3)	Meningitis	Female	Multiple sclerosis	5	8	Recovering
VigiBase 2016 (3)^a^	Listeriosis	Male	Not reported	Unknown	Unknown	Died
VigiBase 2016 (3)	Meningitis	Female	Multiple sclerosis	3	5	Recovered
VigiBase 2016 (3)	Unknown	Female	Multiple sclerosis	5	17	Unknown
VigiBase 2016 (3)	Unknown	Female	Multiple sclerosis	5	23	Unknown
Sanofi Genzyme, data on file VigiBase 2016 (3)	Meningoencephalitis	Female	Multiple sclerosis	5	7	Died
VigiBase 2016 (3)	Meningitis	Female	Multiple sclerosis	5	17	Recovered
VigiBase 2016 (3)	Unknown	Female	Multiple sclerosis	3	8	Recovered
VigiBase 2016 (3)	Unknown	Unknown	Multiple sclerosis	5	9	Unknown
VigiBase 2016 (3)	Septicaemia	Female	Multiple sclerosis	Unknown	Unknown	Unknown
VigiBase 2015 (3)	Unknown	Male	Multiple sclerosis	5	9	Recovered
VigiBase 20 14 (3)	Meningitis	Female	Multiple sclerosis	5	1	Not recovered
Rau 2015 (4)	Meningitis	Female	Multiple sclerosis	5	6	Recovered
Rau 2015 (4)	Meningitis	Female	Multiple sclerosis	5	8	Recovered
Wray 2009 (5)	Meningitis	Female	Multiple sclerosis	3	19	Recovered
Ohm 2009 (6)	Sepsis	Female	Multiple sclerosis	3	13	Not recovered
VigiBase 2010	Meningitis	Male	Unknown	NA	Unknown	Not recovered
VigiBase 2009 (3)	Unknown	Female	B cell lymphoma	NA	Unknown	Died
VigiBase 2010 (3)	Sepsis	Male	Chronic lymphocytic leukemia	NA	Unknown	Unknown
VigiBase 2011	Unknown	Unknown	Chronic lymphocytic leukemia	NA	Unknown	Unknown

^a^No information about the indication for treatment, type of listeriosis or number of infusions is provided at VigiBase for this case

Information in VigiBase comes from a variety of sources, and the likelihood that the suspected adverse reaction is drug-related is not the same in all cases. The information does not represent the opinion of the World Health Organization (3)

Our patient developed clinical symptoms the day after the last infusion of alemtuzumab. Notably, most previous cases of alemtuzumab-associated listeriosis in patients with MS have also presented shortly after treatment. One patient with a poor outcome (reported to VigiBase in 2014) may even have developed symptoms in the beginning of the treatment cycle.

Unlike our patient, it seems that signs of meningitis with headache have been present in most previously reported cases. Thus, headache, neck stiffness, fever, and worsening of pre-existing MS symptoms started at the day of the last infusion in a 47 year old woman [4], whereas a 43 year old man developed fever followed by headache 3 days after the last infusion [4]. The fatal case, a 43 year old woman, was admitted to hospital with low Glasgow Coma Scale score a couple of days after the last infusion of her first alemtuzumab cycle. She developed brain edema and passed away 2 days later. Blood and CSF cultures were positive for listeria (Council for International Organizations of Medical Sciences (CIOMS) report September 16 2016, Sanofi Genzyme, data on file). One of the participants in the CAMMS-223 study, a 36 year woman, was admitted to hospital with fever, abdominal pain and headache 16 days after the last infusion (24 mg) [5], and a 33 year old woman was admitted to hospital with fever and chills 10 days after the final infusion [6].

Two other fatal cases of listeriosis associated with alemtuzumab have been reported to VigiBase. One patient who was treated for lymphoma died in 2009. Another fatal case was reported in December 2016. There are unfortunately no available information about disease characteristics or treatment details for this patient, including whether the treatment indication was MS.

In our patient listeriosis occurred in association with the second treatment cycle. Alemtuzumab-associated listeriosis has previously been reported in MS patients both after the first and the second cycle [4–6]. VigiBase does not provide direct information about treatment cycle. Eleven MS patients have however developed listeriosis in association with five infusions which are used for the first cycle, and five in association with three infusions which are used for later cycles (Table 1). This may simply reflect that not all patients have yet received the second cycle.

*L. monocytogenes* is occasionally present in faeces of healthy immunocompetent persons but does usually not cause disease [7]. The bacteria spread intracellularly, and CD4 and CD 8 T cells are essential for controlling the infection [1]. Alemtuzumab rapidly depletes such cells from the circulation [8], and also reduces the numbers of dendritic cells [9]. Given the long duration of T cell depletion, other factors likely contribute to the aggregation of invasive listeriosis closely after alemtuzumab infusion. Notably, alemtuzumab almost immediately and transiently impairs the release of cytokines from remaining lymphocytes as well as innate immune cells [10]. Such acute and transient effects on both innate and adaptive immunity could explain the peculiar timing of listeria infection to the period immediately after treatment [11].

The SPC for Lemtrada© recommends that patients should avoid ingestion of uncooked or undercooked meats, soft cheeses and unpasteurized dairy products for at least one month after treatment [12]. The incubation period of *L. monocytogenes* varies between 1 to 70 days [1]. Persistence of *L. monocytogenes* after food exposure can be prolonged by corticosteroids, which are now routinely administered prior to alemtuzumab infusions [13]. Our patient had eaten soft cheese and smoked sausage, both known sources of *L. monocytogenes*, 3 days prior to the first infusion and 6 days prior to the debut of the symptoms. She did not consume any such foods during the treatment cycle, and therefore most likely contracted the infection prior to the treatment. One of the other reported cases also consumed raw milk products a few days before the first infusion [6]. We therefore suggest that patients should avoid eating such food items the last weeks prior to alemtuzumab infusion, not only after treatment as currently recommended in the SPC. Investigators have traced outbreaks of listeria infections to a number of food products, including deli meats, hot dogs, soft cheeses (including pasteurised cheeses contaminated after production), celery, sprouts and ice cream [14]. Exposure to *L. moncytogenes* might therefore be difficult to avoid [15].

The present case history highlights that a serious infection can be difficult to distinguish from non-infectious infusion related reactions caused by cytokine release, which may occur up to 24 h after alemtuzumab infusion [16]. Such reactions are less common when infusion of alemtuzumab is preceded by corticosteroids, which are now routinely used. It should however be noted that even when preceded by 1000 mg methylprednisolone alemtuzumab may induce a rapid and transient increase in pro-inflammatory cytokines and acute phase proteins, including c-reactive protein which can rise to septic levels [10]. The differential diagnosis between infectious and non-infectious side effects shortly after alemtuzumab infusions is therefore demanding.

## Conclusion

Physicians and patients should be aware of this potentially lethal side effect of alemtuzumab. The SPC should be revised and advice patients to avoid foods associated with listeria not only after, but also some weeks before treatment with alemtuzumab. The occurrence of listeriosis associated with alemtuzumab should be followed closely, and the need for antibiotic prophylaxis could be considered if prophylactic measures are insufficient.

## Abbreviations

CIOMS: Council for International Organizations of Medical Sciences
SPC: Summary of Product Characteristics
WHO: World Health Organization
